# Supplementary figures and images for: The role of dermis resident macrophages and their interaction with neutrophils in the early establishment of Leishmania major infection transmitted by sand fly bite
Source: PLoS Pathog. 2020 Nov 2;16(11):e1008674. doi: 10.1371/journal.ppat.1008674 (PMC7660907; doi:10.1371/journal.ppat.1008674)

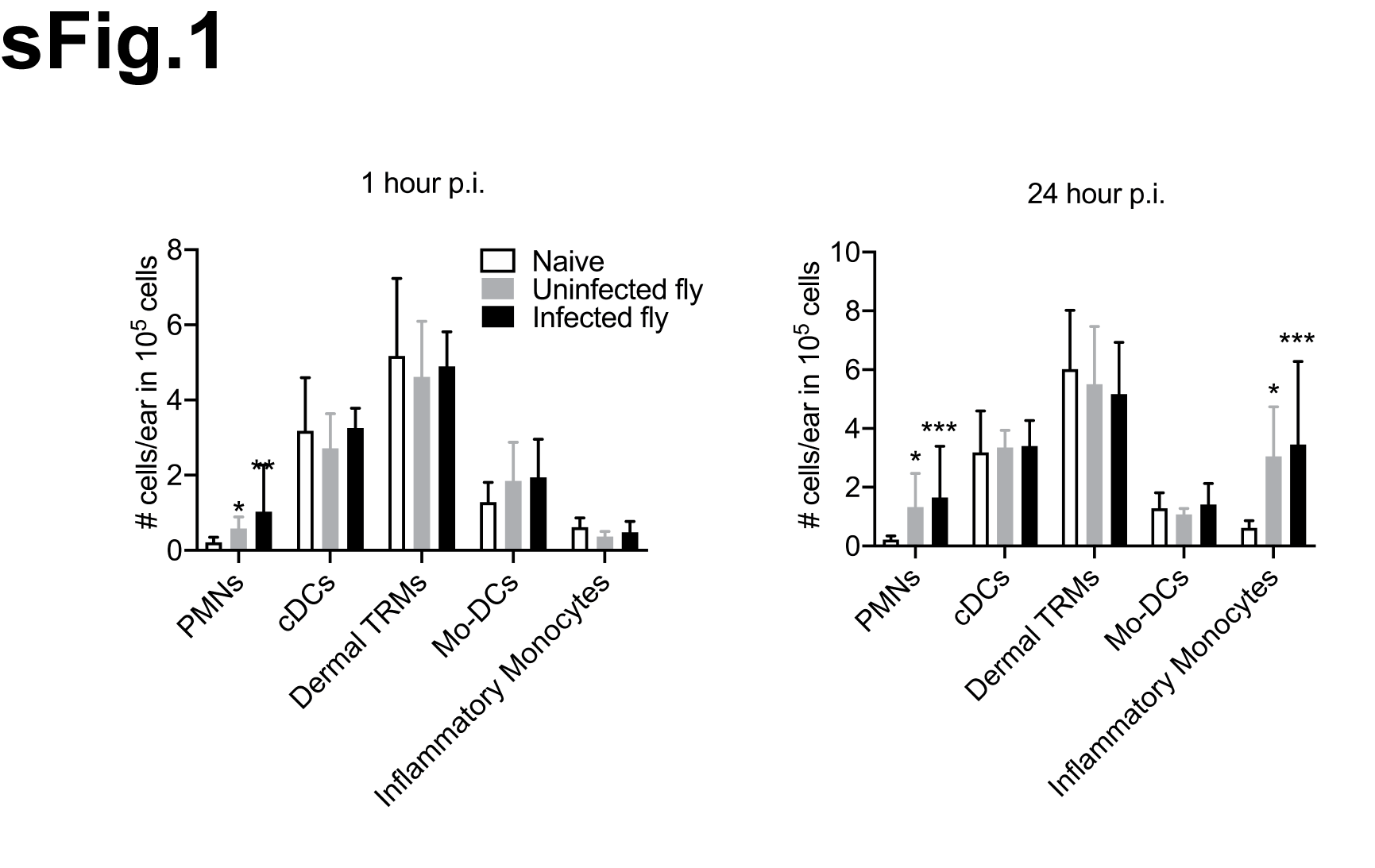

Supplement: S1 Fig — C57BL/6 mice ears were exposed to the bites of either infected or uninfected sandflies and the absolute numbers of myeloid populations in ears were analyzed after 1 and 24 hours by flow cytometry. 6–8 ears per group. Values are means ± SD; *P < 0.05, **P < 0.01, *** P < 0.001. (TIF) [file ppat.1008674.s001.tif]

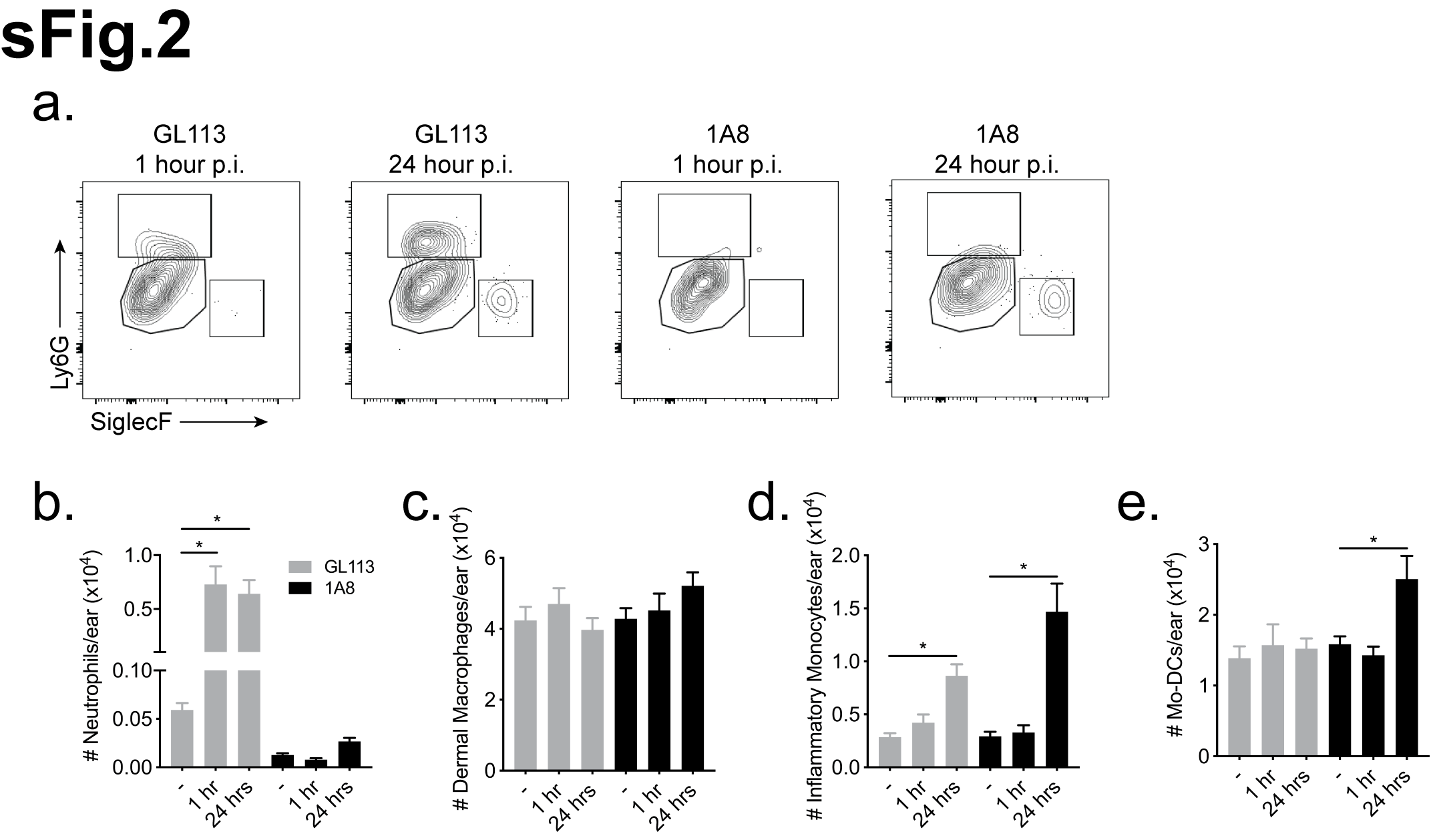

Supplement: S2 Fig — 1A8 treated or control treated C57Bl/6 mice were infected 24 hr later with 2 x 10^5^ RFP+ LmRyn metacyclic promastigotes in the ear dermis. One hr and 24 hrs p.i., the numbers of CD11b+ subsets were determined by flow cytometry. (A) Representative dot plots of ear dermal cells, (B) neutrophils, (C) dermal TRM, (D) inflammatory monocytes and (E) mo-DCs per ear were determined by flow cytometry. Values shown are mean number of cells per ear ± SD, 8–12 ears per group pooled from 2 independent experiments; *P < 0.05. (TIF) [file ppat.1008674.s002.tif]

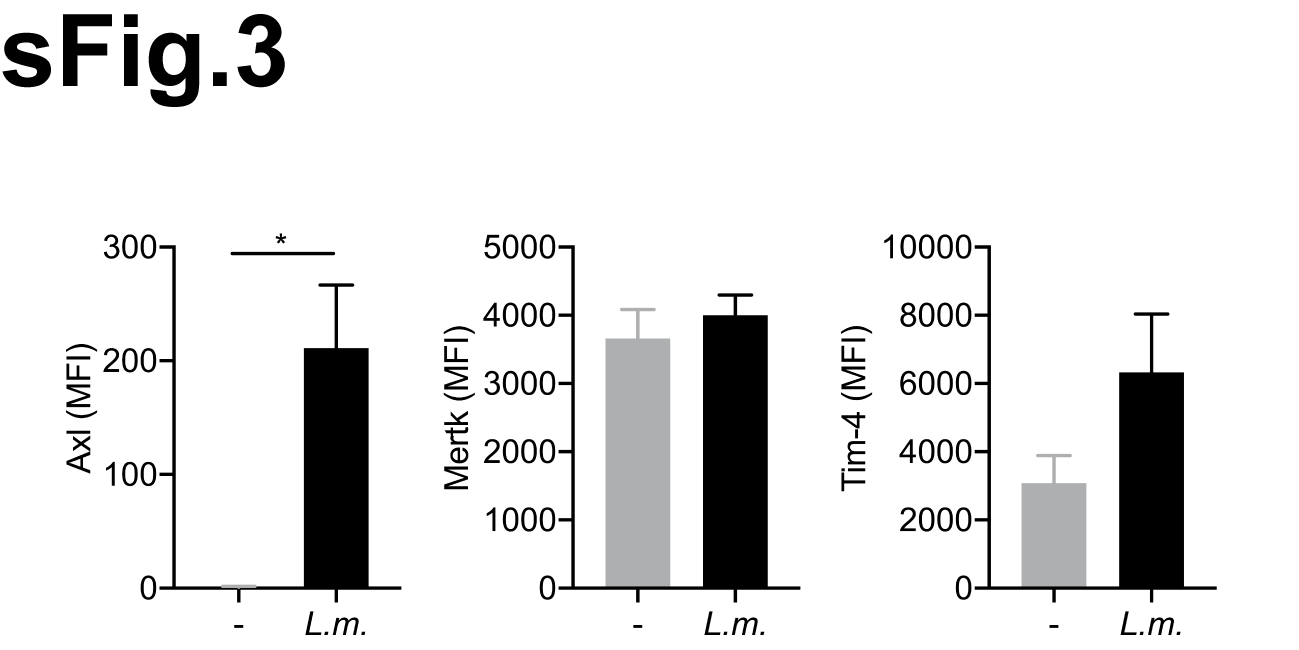

Supplement: S3 Fig — Axl, Tim-4 and MertK expression levels on dermal TRMs from uninfected or infected C57Bl/6 mice at 10 days post-infection with 2 x 10^5^ RFP+ LmRyn metacyclic promastigotes in the ear dermis. Values shown are MFI per ear ± SD, 6 ears per group. (TIF) [file ppat.1008674.s003.tif]

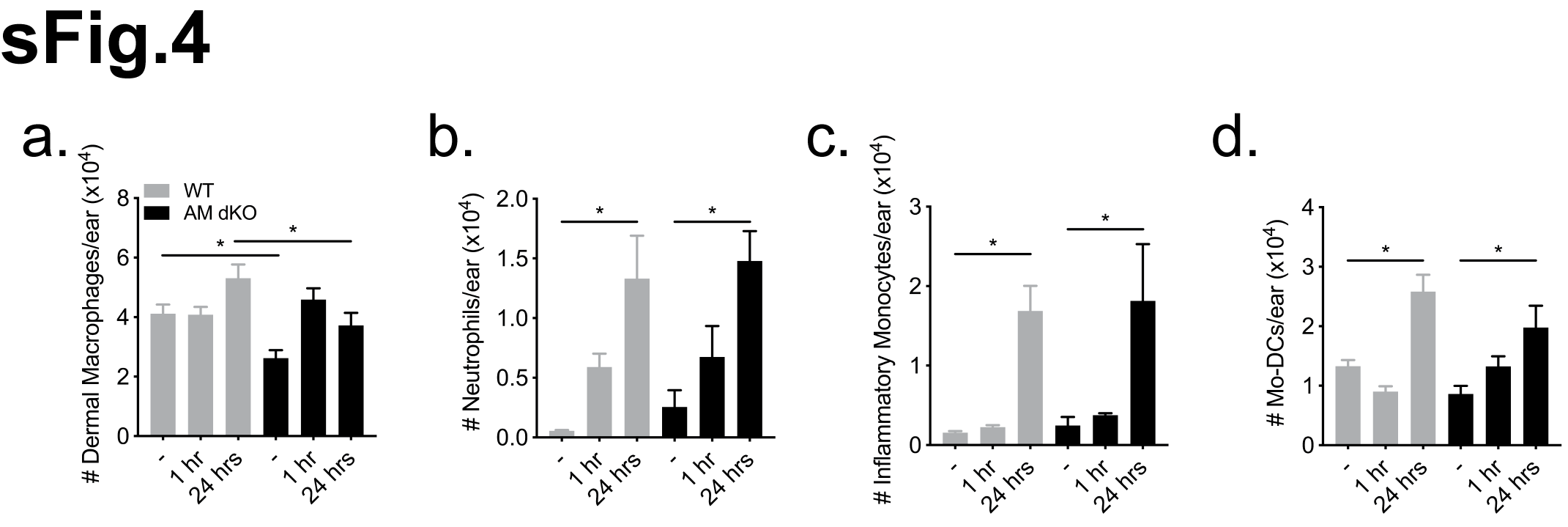

Supplement: S4 Fig — (A-D) WT and Axl-/-Mertk-/- (AM dKO) mice were infected with 2 x 10^5^ RFP+ LmRyn metacyclic promastigotes in the ear dermis. One hr and 24 hrs post infection, the numbers of dermal TRM, neutrophils, inflammatory monocytes and mo-DCs per ear were determined by flow cytometry. Values shown are mean number of cells per ear ± SD, 8–12 ears per group pooled from 2 independent experiments; *P < 0.05. (TIF) [file ppat.1008674.s004.tif]

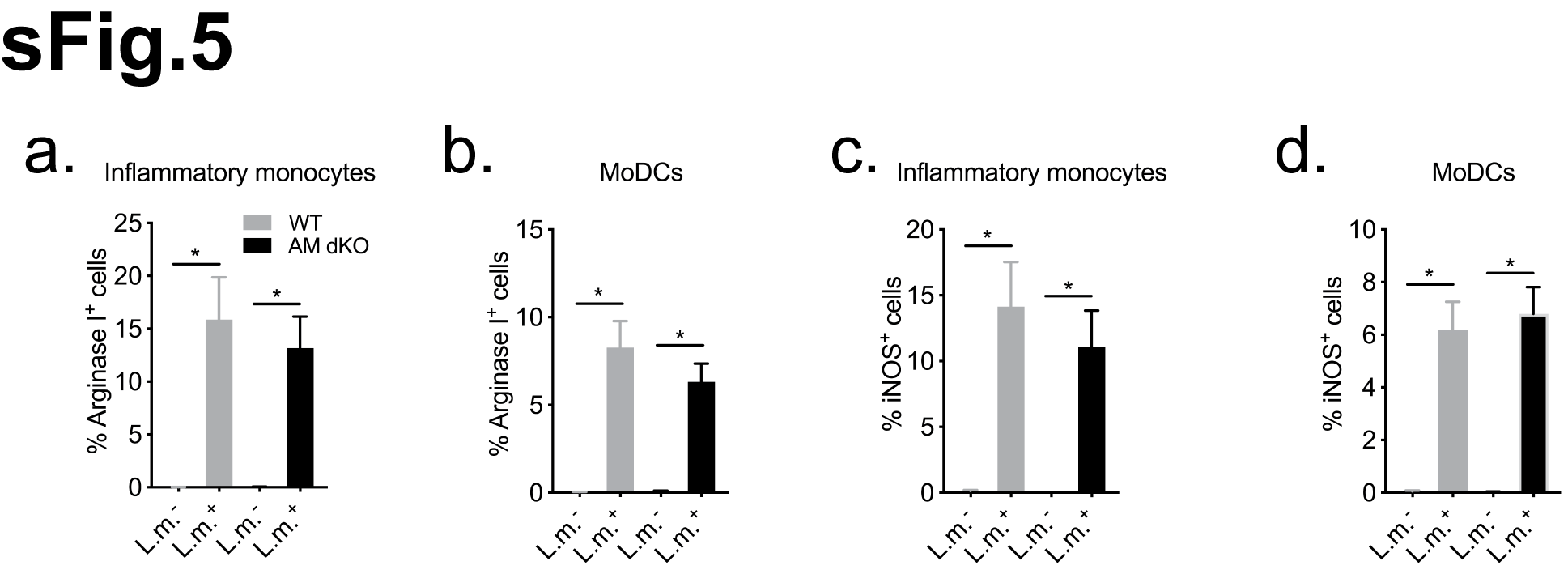

Supplement: S5 Fig — WT (grey bars) and Axl-/-Mertk-/- (AM dKO) (black bars) mice were infected with 2 x 10^5^ RFP+ LmRyn metacyclic promastigotes in the ear dermis. Forty eight hours after infection, frequencies of Arginase 1+ and iNOS+ cells in inflammatory monocytes (A, C) and mo-DCs (B, D) were determined by flow cytometry. Values shown are mean ± SD, 7–12 ears per group pooled from 2 independent experiments; *P < 0.05. (TIF) [file ppat.1008674.s005.tif]

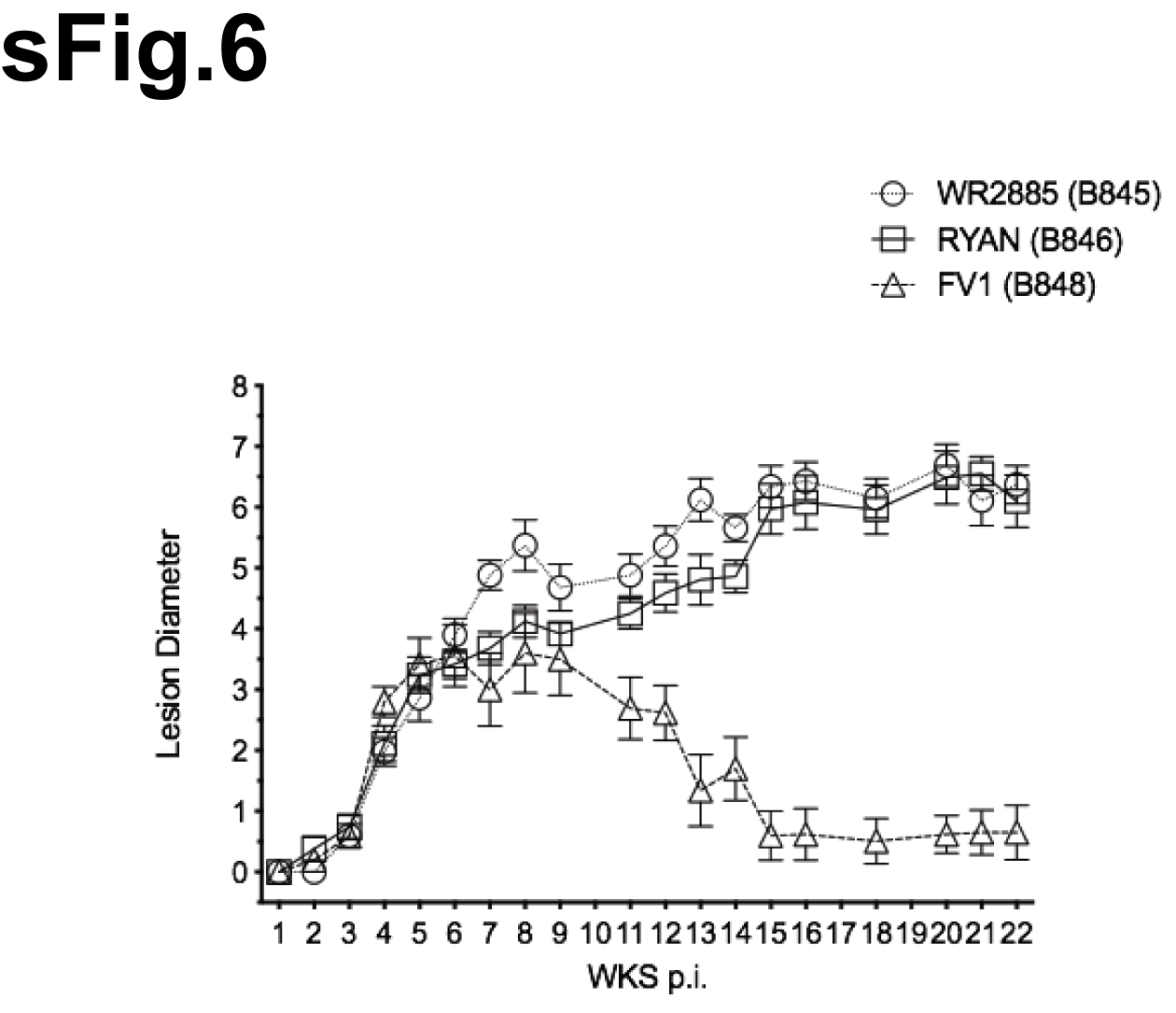

Supplement: S6 Fig — C57BL/6 mice were infected with 103 LmRyn, LmWR2885, or LmFV1 metacyclic promastigotes in the ear dermis. Lesions sizes were measured weekly during 22 weeks infection. Values shown in are means ± SD, 8 ears per group. (TIF) [file ppat.1008674.s006.tif]
